# Synaptic density patterns in early Alzheimer’s disease assessed by independent component analysis

**DOI:** 10.1093/braincomms/fcae107

**Published:** 2024-03-26

**Authors:** Xiaotian T Fang, Nakul R Raval, Ryan S O’Dell, Mika Naganawa, Adam P Mecca, Ming-Kai Chen, Christopher H van Dyck, Richard E Carson

**Affiliations:** Yale PET Center, Yale University School of Medicine, New Haven, CT 06520, USA; Yale PET Center, Yale University School of Medicine, New Haven, CT 06520, USA; Department of Radiology and Biomedical Imaging, Yale University School of Medicine, New Haven, CT 06520, USA; Alzheimer’s Disease Research Unit, Yale University School of Medicine, New Haven, CT 06520, USA; Department of Psychiatry, Yale University School of Medicine, New Haven, CT 06520, USA; Yale PET Center, Yale University School of Medicine, New Haven, CT 06520, USA; Department of Radiology and Biomedical Imaging, Yale University School of Medicine, New Haven, CT 06520, USA; Alzheimer’s Disease Research Unit, Yale University School of Medicine, New Haven, CT 06520, USA; Department of Psychiatry, Yale University School of Medicine, New Haven, CT 06520, USA; Yale PET Center, Yale University School of Medicine, New Haven, CT 06520, USA; Department of Radiology and Biomedical Imaging, Yale University School of Medicine, New Haven, CT 06520, USA; Alzheimer’s Disease Research Unit, Yale University School of Medicine, New Haven, CT 06520, USA; Department of Psychiatry, Yale University School of Medicine, New Haven, CT 06520, USA; Department of Neurology, Yale University School of Medicine, New Haven, CT 06520, USA; Yale PET Center, Yale University School of Medicine, New Haven, CT 06520, USA; Department of Radiology and Biomedical Imaging, Yale University School of Medicine, New Haven, CT 06520, USA

**Keywords:** Alzheimer’s disease, synaptic density, PET, independent component analysis, cognition

## Abstract

Synaptic loss is a primary pathology in Alzheimer’s disease and correlates best with cognitive impairment as found in *post-mortem* studies. Previously, we observed *in vivo* reductions of synaptic density with [^11^C]UCB-J PET (radiotracer for synaptic vesicle protein 2A) throughout the neocortex and medial temporal brain regions in early Alzheimer’s disease. In this study, we applied independent component analysis to synaptic vesicle protein 2A-PET data to identify brain networks associated with cognitive deficits in Alzheimer’s disease in a blinded data-driven manner. [^11^C]UCB-J binding to synaptic vesicle protein 2A was measured in 38 Alzheimer’s disease (24 mild Alzheimer’s disease dementia and 14 mild cognitive impairment) and 19 cognitively normal participants. [^11^C]UCB-J distribution volume ratio values were calculated with a whole cerebellum reference region. Principal components analysis was first used to extract 18 independent components to which independent component analysis was then applied. Subject loading weights per pattern were compared between groups using Kruskal–Wallis tests. Spearman’s rank correlations were used to assess relationships between loading weights and measures of cognitive and functional performance: Logical Memory II, Rey Auditory Verbal Learning Test—long delay, Clinical Dementia Rating sum of boxes and Mini-Mental State Examination. We observed significant differences in loading weights among cognitively normal, mild cognitive impairment and mild Alzheimer’s disease dementia groups in 5 of the 18 independent components, as determined by Kruskal–Wallis tests. Only Patterns 1 and 2 demonstrated significant differences in group loading weights after correction for multiple comparisons. Excluding the cognitively normal group, we observed significant correlations between the loading weights for Pattern 1 (left temporal cortex and the cingulate gyrus) and Clinical Dementia Rating sum of boxes (*r* = −0.54, *P* = 0.0019), Mini-Mental State Examination (*r* = 0.48, *P* = 0.0055) and Logical Memory II score (*r* = 0.44, *P* = 0.013). For Pattern 2 (temporal cortices), significant associations were demonstrated between its loading weights and Logical Memory II score (*r* = 0.34, *P* = 0.0384). Following false discovery rate correction, only the relationship between the Pattern 1 loading weights with Clinical Dementia Rating sum of boxes (*r* = −0.54, *P* = 0.0019) and Mini-Mental State Examination (*r* = 0.48, *P* = 0.0055) remained statistically significant. We demonstrated that independent component analysis could define coherent spatial patterns of synaptic density. Furthermore, commonly used measures of cognitive performance correlated significantly with loading weights for two patterns within only the mild cognitive impairment/mild Alzheimer’s disease dementia group. This study leverages data-centric approaches to augment the conventional region-of-interest–based methods, revealing distinct patterns that differentiate between mild cognitive impairment and mild Alzheimer’s disease dementia, marking a significant advancement in the field.

## Teaser

Fang *et al*. utilize synaptic density PET imaging and independent component analysis to uncover distinct synaptic patterns in Alzheimer’s disease. These patterns discern among cognitively normal, mild cognitive impairment and mild Alzheimer’s disease dementia groups and correlate significantly with widely recognized cognitive function measures, underscoring the technique’s diagnostic relevance.

## Graphical Abstract

**Graphical Abstract:**
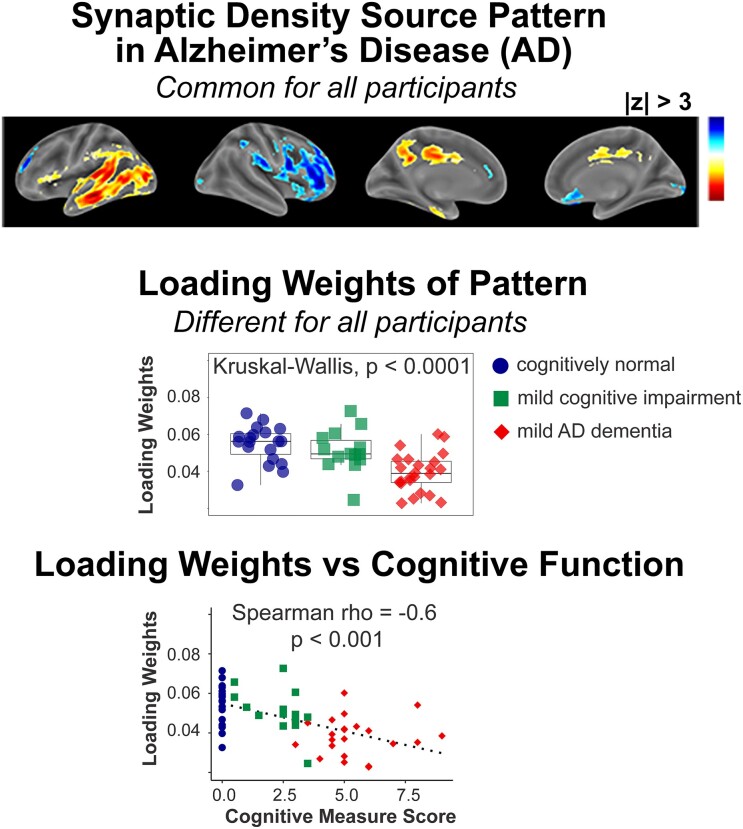

## Introduction

Synapse loss is an early pathophysiological event in Alzheimer’s disease and shows robust association with cognitive impairment.^1-4^ However, these findings were largely derived from autopsy studies conducted during later stages of disease. With the development of [^11^C]UCB-J (and analogues [^18^F]SynVesT-1, [^18^F]SynVesT-2, [^11^C]UCB-H and [^11^C]UCB-A) PET, a recently developed non-invasive imaging method targeting synaptic vesicle protein 2A (SV2A), synaptic density can now be assessed *in vivo.*^5-10^ SV2A is ubiquitously expressed in all pre-synaptic axon terminals, making it a potential *in vivo* marker for synaptic density.^7,11,12^ Previously, [^11^C]UCB-J PET studies reported decreased SV2A binding in medial temporal and neocortical regions in early Alzheimer’s disease compared with cognitively normal (CN) participants.^13,14^ While some studies have shown associations in specific regions or under certain conditions, the overall relationship between synaptic density and cognitive performance, especially when excluding the CN group, has been less clear.^13-15^ A study by Mecca *et al*.^16^ demonstrated a significant positive association between global synaptic density and global cognition and performance in five individual cognitive domains using pre-defined regions of interest (ROIs) within Alzheimer’s disease participants.

Conventional PET image analysis involves either *a priori*–established ROIs typically based on anatomical features or voxel-based analysis whereby spatially standardized images are analysed on the voxel level. However, more sophisticated analytical approaches exist (e.g. cluster analysis), which may be more sensitive and thus more appropriate, especially in Alzheimer’s disease. The method applied here, source-based morphometry, is a biomedical image analysis approach, which uses a data-driven method, spatial independent component analysis (ICA), to decompose mixed signal image information into maximally independent sources (images) of common variance. This method determines these variance patterns and subject-specific loading weights without information about subject group membership. Statistical analysis can then be used to identify which sources distinguish healthy controls from those with pathological conditions.^17^ This has previously been used to investigate spatial patterns of ^18^F-fluorodeoxyglucose (FDG) PET data,^18,19^ grey matter atrophy related to healthy ageing,^20^ schizophrenia^17^ and multiple sclerosis,^21^ as well as to identify independent sources of synaptic density in healthy controls.^22^

We aimed to investigate whether changes in synaptic density in Alzheimer’s disease follow specific patterns using this data-driven approach. Additionally, we investigated whether these patterns were associated with clinical and cognitive measures.

## Material and methods

### Subjects and study design

Participants provided written informed consent prior to participation as approved by the Yale University Human Investigation Committee. The study cohort consisted of 19 CN participants and 38 participants with Alzheimer’s disease [14 with mild cognitive impairment (MCI) and 24 with mild Alzheimer’s disease dementia] aged 50–85 years. A full description regarding recruitment and eligibility of the cohort can be found here.^14^ In brief, recruited individuals with mild Alzheimer’s disease dementia were required to meet diagnostic criteria for probable dementia,^23^ have a global Clinical Dementia Rating (CDR) score of 0.5–1.0 and have a Mini-Mental State Examination (MMSE) score <26. Individuals with MCI were included if they met diagnostic criteria for amnestic MCI,^24^ had a global CDR score of 0.5, and had an MMSE score of 24–30. Mild Alzheimer’s disease dementia and MCI participants were required to demonstrate impairments in episodic memory, as measured by a Logical Memory II (LMII) score 1.5 SD below an education-adjusted norm. CN participants were required to have a CDR score of 0, an MMSE score >26 and a normal education adjusted LMII score. The Rey Auditory Verbal Learning Test (RAVLT) was also administered, and the long delay was used to generate an episodic memory score. The presence of amyloid-β accumulation was determined as previously described:^13,14^ all participants received a PET scan with [^11^C]Pittsburgh Compound B ([^11^C]PiB) and were required to be negative for CN participants and positive for MCI/mild Alzheimer’s disease dementia participants. Subjects and imaging data used in the current work were highly overlapping with the cohort described in the previous study.^14^

### Brain imaging

Participants underwent T_1_-weighted MRI scans on a 3-T Trio (Siemens). PET scans were acquired on the High-Resolution Research Tomograph (Siemens), which acquires 207 slices (1.2 mm slice separation) with a reconstructed image resolution (full width at half maximum) of ∼3 mm. Prior to each PET scan, a 6-min transmission scan was performed for attenuation correction. PET data were acquired in list mode. Dynamic scans were acquired for 60 min after i.v. bolus administration of [^11^C]UCB-J (572.2 ± 188.9 MBq, min 157.6–max 761.8 MBq). Dynamic PET data were reconstructed with corrections for attenuation, normalization, scatter, randoms and dead time using the motion-compensation OSEM list-mode algorithm for resolution-recovery reconstruction algorithm.^25^ Event-by-event motion correction was included in the reconstruction by tracking motion with a Polaris Vicra optical tracking system (NDI Systems) using reflectors mounted on a swim cap worn by subject.

For each subject, motion-corrected dynamic PET data were co-registered to an early summed PET image (0–10 min after [^11^C]UCB-J administration) using a six-parameter mutual information algorithm (FSL-FLIRT). The summed PET image was co-registered to the individual’s T_1_-weighted MR image (six-parameter rigid registration), followed by a non-linear transformation to the automated anatomical labelling template in the Montreal Neurological Institute space using BioImage Suite.^26^

### Kinetic modelling

For a full description of the PET kinetic analysis, see our previous study.^14^ In brief, for [^11^C]UCB-J image analysis, parametric binding potential (BP_ND_) images were first generated using the simplified reference tissue model-2 step with the centrum semiovale as reference region.^27^ Simplified reference tissue model-2 step was performed using a fixed global $k_{2}^{\prime }$ value (reference region clearance rate constant) of 0.027 min^−1^, which was estimated as a *k*_2_ population average of the centrum semiovale obtained with one-tissue compartment modelling from a previous subject group that underwent arterial blood sampling (Yale PET Center dataset). Lastly, parametric distribution volume ratio images with a whole cerebellum reference region (DVR_Cb_) were computed from BP_ND_ images as (BP_ND_ +1)/(BP_ND, Cb_ + 1). The whole cerebellum has previously been validated as a reference region for [^11^C]UCB-J in Alzheimer’s disease studies, demonstrating similar values of cerebellar distribution of volume (*V*_T_) between Alzheimer’s disease and CN participants.^14^

### Analysis of synaptic density patterns: source-based morphometry

Parametric [^11^C]UCB-J DVR_Cb_ images in the Montreal Neurological Institute template space were smoothed using a Gaussian kernel with a full width at half maximum of 8 mm prior to further analysis. Source-based morphometry was performed on the spatially normalized and smoothed parametric DVR_Cb_ maps using the GIFT toolbox (GIFTv4.0b; trendscenter.org/software/gift/). For each subject, voxel-wise DVR_Cb_ values were concatenated into a single row vector, and the vectors of all subjects were concatenated into a 2D matrix (number of subjects × number of voxels). Analyses were constrained to a voxel mask containing the whole brain, and the average subject-wise DVR_Cb_ value was removed from the data by subtracting the mean from each row of this matrix. Prior to decomposition, principal component analysis was performed to reduce dimensionality of the data. In this study, the number of components was set at 18 based on previous analyses in order to extract 18 independent components (ICs).^22^ Spatial ICA was then performed using the Infomax algorithm,^28^ which decomposed the data into a mixing matrix (number of subjects × number of components) and a source matrix (number of components × number of voxels). Each row of the mixing matrix contains a subject’s loading weights (unitless) of corresponding synaptic density ([^11^C]UCB-J DVR_Cb_) patterns (i.e. sources) and can be interpreted as the extent to which a pattern is present in an individual subject. Source matrix row values represent the corresponding synaptic density patterns. For visualization, each source was scaled to unit standard deviation (*Z* map) and thresholded at a value of |*Z*| > 3.

Larger loading weights for a subject indicate a stronger presence of the corresponding synaptic density pattern. If loading weights are lower for MCI/mild Alzheimer’s disease dementia participants compared with CN individuals, this can be interpreted as indicating a greater loss of synaptic density in the MCI/mild Alzheimer’s disease dementia group relative to global synaptic density.

### Statistical analysis

Statistical analyses were performed in R 4.0.2. Demographic and clinical characteristics comparisons were made using χ^2^ test for categorical variables and unpaired *t*-tests for continuous variables. Kolmogorov–Smirnov tests and visual inspection of the histogram were used to assess normality of the variables. Variables were not normally distributed; thus, the Kruskal–Wallis test was used. The Benjamini–Hochberg procedure was performed to control the false discovery rate (FDR) for multiple comparisons [18 comparisons for ICs between CN, MCI, and mild Alzheimer’s disease dementia diagnostic groups]. The relationship between loading weights and episodic memory or global memory function was assessed using Spearman’s rank correlation, controlling the FDR for multiple comparisons using the Benjamini–Hochberg procedure in two analyses: (i) in the MCI and mild Alzheimer’s disease dementia groups only and (ii) in the full cohort including the CN. Tests were two-tailed, and *P* < 0.05 was set as the threshold for significance.

## Results

### Demographics and characteristics

A total of 57 participants were included, consisting of 14 with amnestic MCI due to Alzheimer’s disease, 24 with mild Alzheimer’s disease dementia and 19 CN participants. Diagnostic groups were well balanced for sex (χ^2^  _=_ 2.55, *P* = 0.28) and age [*F*(2,54) = 0.30, *P* = 0.74], though CN participants had more years of education than participants with mild dementia (CN: 17.7 ± 2.1, mild dementia: 15.8 ± 2.4, *P* = 0.02; Supplementary Table 1). Both the MCI and the mild dementia participants displayed typical clinical characteristics of cognitive and functional impairment [MMSE: MCI: 26.3 ± 2.9, mild dementia: 21.5 ± 3.0; CDR sum of boxes (CDR-sb): MCI: 2.3 ± 1.0, mild dementia: 5.3 ± 1.5], and CN participants demonstrated no cognitive deficits (MMSE: 29.2 ± 1.1, CDR-sb: 0 ± 0; Supplementary Table 1). Further demographic and clinical information are presented in Supplementary Table 1.

### Patterns of synaptic density

**Figure 1:**
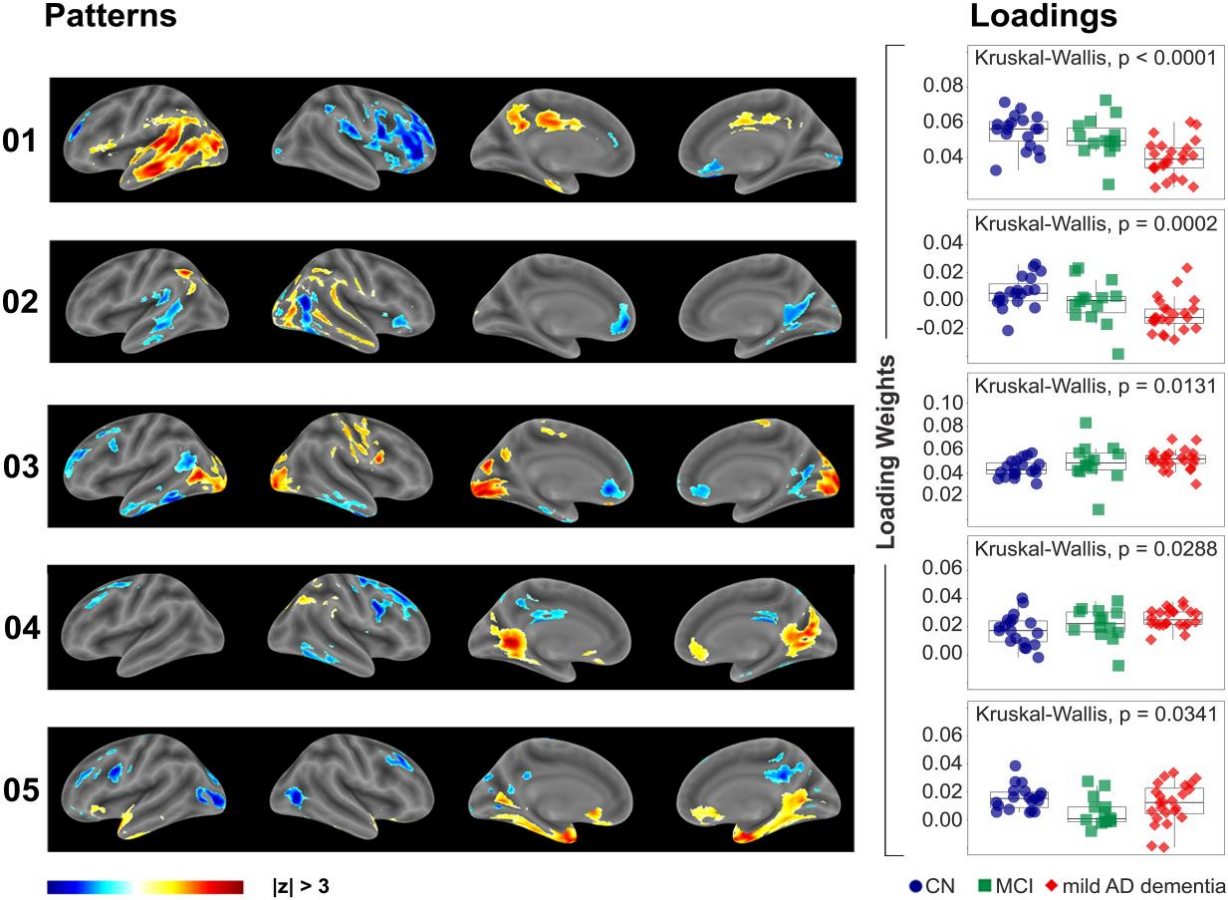
**Significant ICA patterns and loading weights.** Synaptic density spatial patterns were identified by ICA. Patterns were *Z*-scores transformed, and the Kruskal–Wallis comparison of the loading weights determined 5 (out of 18) patterns with significant diagnostic group differences, depicted in Rows 1–5. Each row displays co-varying spatial density patterns with |*Z*| > 3 and corresponding loading weights.

From the spatially normalized and smoothed parametric [^11^C]UCB-J DVR_Cb_ images, 18 synaptic density patterns were extracted by principal components analysis. Kruskal–Wallis comparison of the synaptic density loading weights between diagnostic groups demonstrated five patterns that differed significantly between diagnostic groups [Pattern 1: *F*(2,57) = 18.8, *P* < 0.0001; Pattern 2: *F*(2,57) = 17.0, *P* = 0.0002; Pattern 3: *F*(2,57) = 8.7, *P* = 0.0131; Pattern 4: *F*(2,57) = 7.1, *P* = 0.0288; and Pattern 5: *F*(2,57) = 6.8, *P* = 0.0341; Fig. 1, Table 1]. The clusters encompassing the patterns are reported in Table 1, ordered by hemisphere and maximum *Z*-value, as well as the group-wise comparisons of the corresponding loading weights. Most of these patterns show distinct localization as well as symmetry between hemispheres. However, in a few patterns, there are major clusters present in only one hemisphere, e.g. the largest positive cluster in Pattern 1 is primarily located in the temporal lobe of the left hemisphere, as well as displaying a large negative cluster in the right frontal lobe. Participants with mild Alzheimer’s disease dementia showed lower loading weights compared with CN participants for Patterns 1, 2, 3 and 4 (Fig. 1, Table 1). Additionally, loading weights for mild Alzheimer’s disease dementia participants were significantly lower compared with MCI participants for Patterns 1 and 2. Lastly, Pattern 5 displayed lower bindings for MCI participants compared with CN.

**Table 1:** Synaptic density patterns identified by ICA and group-wise comparisons of corresponding loading weights

Summary	*Z*-score	Size (voxels)	Significant differences
Pattern 1			
L Mid temporal, extending into mid occipital	10.001	15 131	Mild Alzheimer’s disease dementia versus CN**** Mild Alzheimer’s disease dementia versus MCI**
R Inf tri, inf sup frontal	−8.804	6525	
L Mid frontal	−7.664	763	
L Post-cingulate, ext. into R mid cingulate	7.526	888	
R Rolandic oper	−7.173	935	
L Mid cingulate	6.323	1119	
R Sup temporal pole	−5.408	873	
L/R Cerebellum	−5.329	591	
R Inf occipital	−4.583	703	
Pattern 2			
R Inf temporal gyrus	10.553	16 952	Mild Alzheimer’s disease dementia versus CN**** Mild Alzheimer’s disease dementia versus MCI**
R Mid/inf temporal gyrus, ext. into lingual	10.517	1946	
L Angular	10.06	806	
L Anterior cingulate	−7.787	568	
L Mid/sup temporal	−6.796	1513	
L Inf temporal gyrus	6.109	1269	
R Calcarine, ext. into lingual, parahippocampal	−6.040	1152	
R Insula, putamen	−5.674	614	
Pattern 3			
L Inf/mid temporal	−10.507	4276	MCI versus CN* Mild Alzheimer’s disease dementia versus CN**
L Cerebellum	8.750	16 802	
L Mid frontal	−7.409	3464	
R Pre-central	6.619	1628	
R Calcarine	−6.145	727	
R Paracentral lobule	5.422	621	
Pattern 4			
L Lingual	12.646	6309	Mild Alzheimer’s disease dementia versus CN**
R Cerebellum	10.933	9523	
R Sup frontal	−8.175	2815	
L Rectus	7.745	850	
R Inf temporal	−7.783	788	
R Precuneus	−5.732	1002	
L Precuneus	−5.448	892	
R Pallidum	−4.247	2134	
Pattern 5			
L/R Sup temporal pole, ext. into parahippocampal	13.756	20 806	MCI versus CN** Mild Alzheimer’s disease dementia versus MCI*
R Mid occipital	−8.676	814	
L Mid occipital	−7.720	1525	
L Pre-central	−7.105	526	
L Lingual	−6.826	1414	
L Mid frontal	−6.639	1082	
R Mid/sup frontal	−6.217	1054	
L Inf tri frontal	−5.688	963	

Clusters with |*Z*| > 3.0 and size > 500 voxels are listed, ordered by maximum absolute *Z*-value. CN, control. *Non-FDR corrected indicates significant group differences in loading weights: *P < 0.05, **P < 0.01 and ****P < 0.0001.

After applying the Benjamini–Hochberg procedure to the Kruskal–Wallis comparison, only two patterns (Patterns 1 and 2) had loading weights that remained statistically significant between diagnostic groups. For Pattern 1, participants with mild Alzheimer’s disease dementia had significantly lower loading weights compared with both MCI (*P* = 0.0022) and CN participants (*P* < 0.0001). Similarly, for Pattern 2, participants with mild Alzheimer’s disease dementia had significantly lower loading weights than MCI (*P* = 0.031) and CN participants (*P* < 0.0001; Fig. 1).

### Association between cognitive performance and synaptic density patterns in MCI and mild dementia

**Figure 2:**
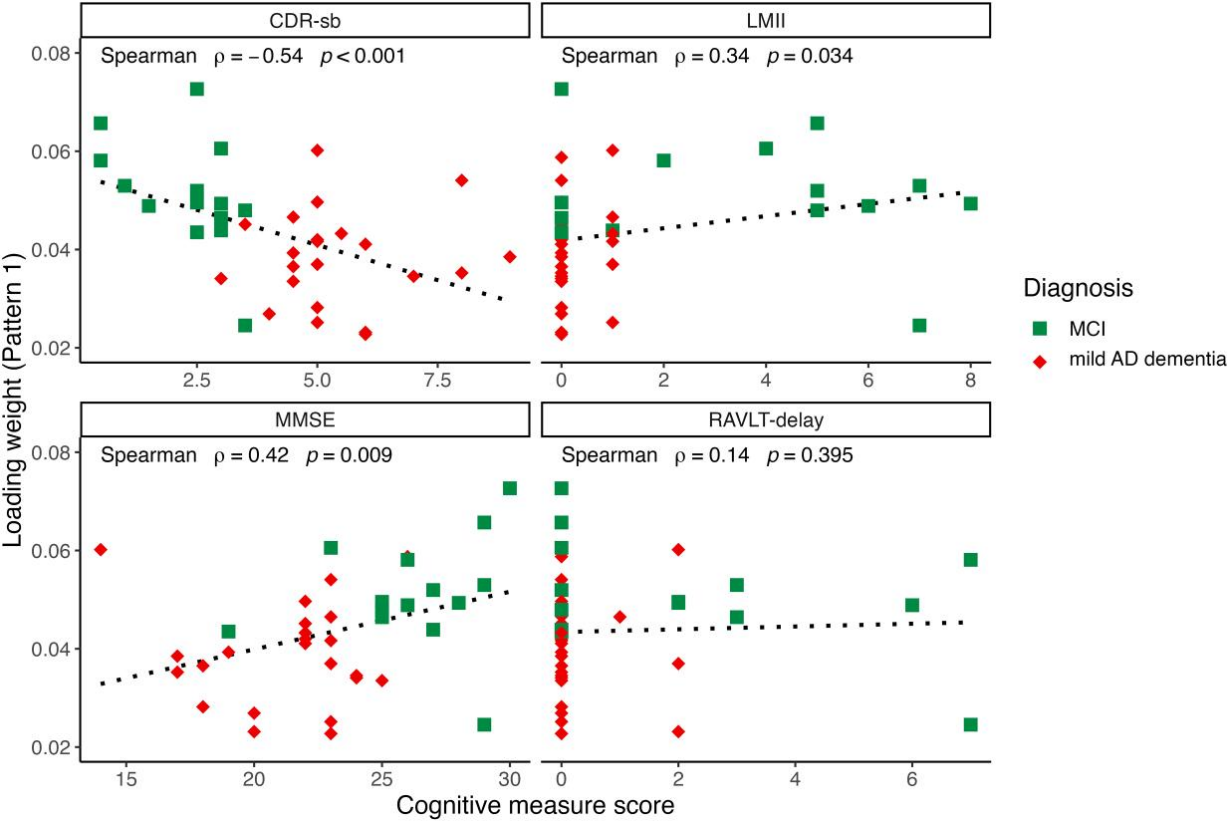
**Pattern 1 loading weights versus cognition.** Correlations of Pattern 1 subject loading weights and measures of cognitive and functional performance in MCI and mild Alzheimer’s disease dementia participants. Within the disease group only, scatter plots depict significant relationships between loading weights and CDR-sb, LMII and MMSE scores. Spearman’s correlations were calculated, and following FDR correction, only the relationship between the loading weights with CDR-sb and with MMSE remained statistically significant. The dashed line represents linear regression line.

**Figure 3:**
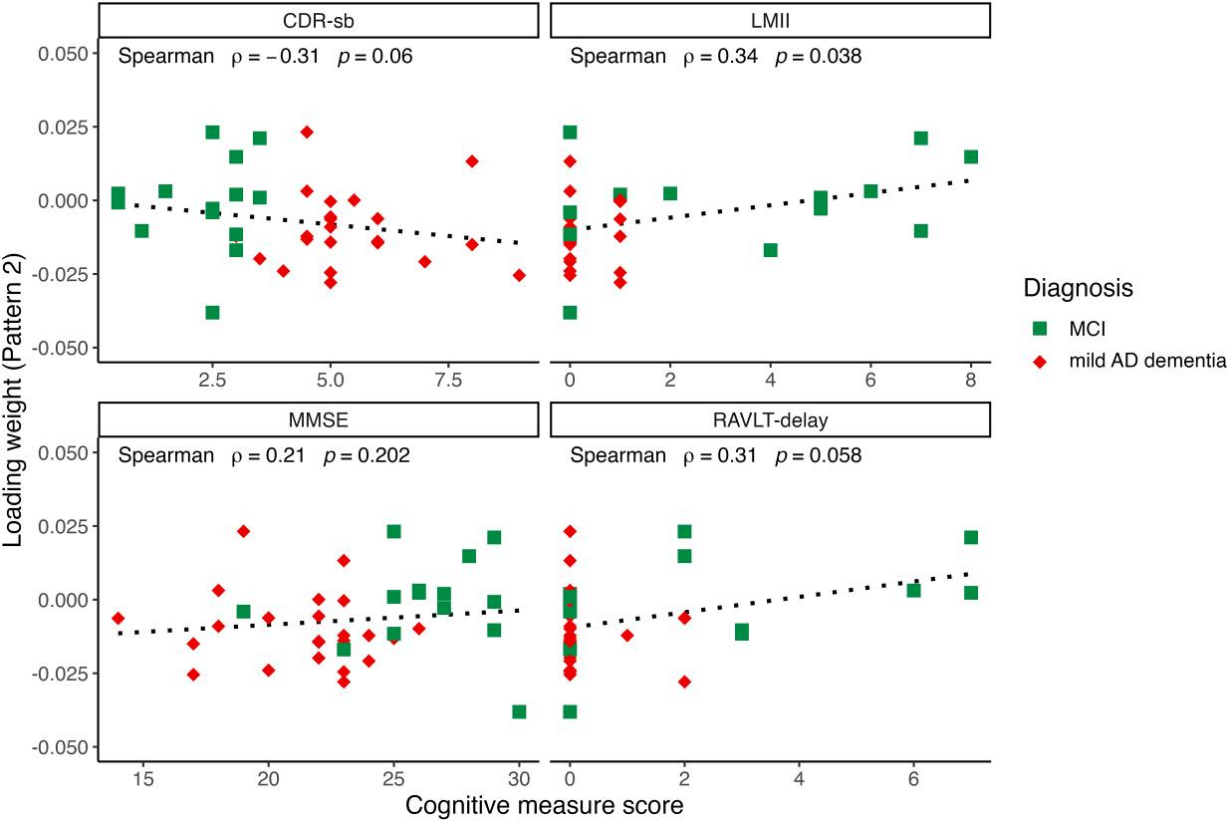
**Pattern 2 loading weights versus cognition.** Correlations of Pattern 2 subject loading weights and measures of cognitive and functional performance in MCI and mild Alzheimer’s disease dementia participants. Within the disease group only, scatter plots depict significant relationships between loading weights and LMII score. Spearman’s correlations were calculated without correction for multiple comparisons. The dashed line represents linear regression line.

We investigated the association of the loading weights of each synaptic density pattern with functional and cognitive measures, including CDR-sb, LMII, MMSE and Rey Auditory Verbal Learning Test (RAVLT)—long delay scores using Spearman’s rank correlations. To assess the relationship with severity of cognitive impairment within disease state, we excluded CN participants and subsequently analysed only the MCI and mild Alzheimer’s disease dementia subject loading weights. Within this symptomatic cohort, statistically significant correlations were found between the loading weights for Pattern 1 and CDR-sb (*r* = −0.54, *P* < 0.001), MMSE (*r* = 0.48, *P* = 0.009) and LMII score (*r* = 0.44, *P* = 0.034; Fig. 2). In addition, statistically significant associations were observed between the loading weights for Pattern 2 and LMII score (*r* = 0.34, *P* = 0.038; Fig. 3). Following FDR correction, only the relationship between the loading weights for Pattern 1 with CDR-sb (*r* = 0.54, *P* = 0.003) and with MMSE (*r* = 0.48, *P* = 0.036) remained statistically significant.

Additionally, statistically significant correlations were found within the overall sample (i.e. including CN, MCI and mild Alzheimer’s disease dementia participants) between the loading weights for Pattern 1 and CDR-sb (*r =* −0.60, *P* < 0.001), MMSE (*r* = 0.50, *P* < 0.001), LMII (*r* = 0.44, *P* < 0.001) and RAVLT-delay (*r* = 0.39, *P* = 0.003). Significant correlations were also found between the loading weights of Pattern 2 and CDR-sb (*r* = −0.51, *P* < 0.001), MMSE (*r* = 0.42, *P* = 0.001), LMII (*r* = 0.53, *P* < 0.001) and RAVLT-delay (*r* = 0.56, *P* < 0.001). These findings are reported in Supplementary Figs 1 and 2. All of these analyses survived FDR correction for multiple comparisons.

## Discussion

We used the ICA of [^11^C]UCB-J PET to identify brain patterns of synaptic density and to explore the relationship between measures of cognitive performance and pattern strength in individuals with normal cognition, MCI and mild Alzheimer’s disease dementia. We focused on the brain patterns of covariance in synaptic density with subject loading weights that showed group-wise differences between diagnostic groups. Furthermore, we observed significant associations between measures of cognitive and functional performance and subject loading weights for two of these patterns with only the mild Alzheimer’s disease dementia and MCI diagnostic groups included.

### Brain patterns of synaptic density

In this study, we identified brain patterns of co-varying synaptic density in a cohort of CN, MCI and mild Alzheimer’s disease dementia participants. Previously, we identified and validated similar brain patterns of co-varying synaptic density in a study consisting of CN participants only, with a wider age range.^21^ While there is some overlap of CN participants, overall, we obtained consistent and similar brain patterns between these two studies. Specifically, out of the original 13 identified and validated ICs in the CN study, 9 ICs were replicated (i.e. visually similar and encompassed same brain regions) in this study (ICs 01–06, IC 08, IC 09 and IC 13). Given that significant pathology occurs in the brains of participants with Alzheimer’s disease, it was unclear whether these brain patterns would be replicated.

Brain patterns with subject loading weights that differed between diagnostic groups consisted of the left lateral temporal, left precuneus and bilateral posterior cingulate gyrus (Pattern 1); right temporal cortices (Pattern 2), lateral/medial occipital, cerebellar and right pre-central (Pattern 3); lingual, right medial orbital frontal and cerebellar (Pattern 4); and a medial temporal pattern extending into the parahippocampal (i.e. entorhinal and perirhinal cortices, Pattern 5). Interestingly, the brain pattern consisting of the entorhinal and perirhinal regions (Pattern 5) had loading weights that showed a group-wise reduction in the MCI group versus both the CN and the mild Alzheimer’s disease dementia group. The regions detected altogether in these brain patterns (1–5) are largely in line with previous MRI studies. Taken together, the identified patterns encompass the medial and inferior temporal lobes including the hippocampus and entorhinal cortices. These regions are known to be some of the earliest affected in the progression of Alzheimer’s disease pathology,^29^ and ROI-based studies have shown that medial temporal measurements can provide the best discrimination between Alzheimer’s disease and CN groups.^30,31^ Pattern 1 also includes the cingulate gyrus, a region which is intimately connected to the hippocampus and entorhinal cortices.^32^

Initial SV2A PET studies in Alzheimer’s disease reported significant reductions in hippocampal synaptic density.^13^ However, a follow-up study demonstrated significant decreases in synaptic density in the entorhinal cortex, hippocampus, amygdala, lateral temporal, pre-frontal, lateral parietal and pericentral regions in Alzheimer’s disease participants.^14^ These findings are largely in line with our current study, which uses a highly overlapping dataset. However, we did not observe the frontal cortex or parietal cortex within our significant brain patterns. A possible explanation for this may be that the changes in synaptic density in these regions did not occur in a consistent manner such that it did not register as a covariance within our brain patterns.

### Data-driven algorithms help improve diagnostic accuracy

A major novelty of this study is the data-driven nature of the applied analysis method, where we make no *a priori* assumptions on the data e.g. subject info/demographics or use of pre-defined ROIs. Nonetheless, brain covariance patterns were identified that are spatially similar to early Braak staging of Alzheimer’s disease. Similarly, there have been previous studies applying comparable data-driven automated image–based algorithms to identify spatial patterns of [^18^F]FDG PET to discriminate between subtypes of parkinsonism (idiopathic Parkinson’s disease, multiple systems atrophy and progressive supranuclear palsy), improving diagnostic accuracy (80%) compared with conventional clinical diagnosis (66%).^33^

This cohort, which has been previously studied using pre-defined template regions,^16^ showed aberrant synaptic patterns in similar brain regions, namely temporal and cingulate cortices, but not frontal or occipital cortices. A parallel study using principal component analysis (PCA) identified distinct patterns across ROIs, with significant contributions from subcortical and parieto-occipital cortical regions.^34^ These PCA findings, particularly the positive correlation of component scores with cognitive domains and the negative correlation with global amyloid deposition, provide a complementary perspective to our ICA results.^34^ Of major interest is that the current study was able to discriminate between MCI and mild Alzheimer’s disease dementia groups, as subject loading weights were significantly different between MCI and mild Alzheimer’s disease dementia groups for Patterns 1 (mild Alzheimer’s disease dementia versus MCI, *P* = 0.0022) and 2 (*P* = 0.031; Fig. 1). Comparatively, no such discriminative effect was accomplished using pre-defined (composite) ROIs,^14^ suggesting that this data-driven method may be more sensitive to early synapse–related pathology in Alzheimer’s disease.

### Strength of brain pattern encompassing temporal and cingulate cortices is related to cognitive performance

Building on our recent work,^34^ we explored the relationship between brain patterns of synaptic density and clinical measures of cognitive performance in Alzheimer’s disease. We found correlations between subject loading weights for several patterns and outcome measures of cognitive performance, even when excluding the CN participants. After adjusting for multiple comparisons, the pattern encompassing temporal and cingulate cortices showed associations with CDR-sb and MMSE. This suggests potential links between synaptic density in these regions and cognitive performance. Previous *post-mortem* electron microscopy studies have found significant decline in synaptic levels in the posterior cingulate gyrus of individuals in the early stages of Alzheimer’s disease compared with CN individuals, with MCI patients exhibiting synaptic numbers that were between Alzheimer’s disease and CN cohorts.^32^ Additionally, the cingulate gyrus is strongly connected with the hippocampal/entorhinal cortex regions, areas of demonstrated robust and early synaptic loss in AD^4,35,36^ and part of the medial temporal memory circuit. Synapse levels in the temporal gyrus are also lower to a similar degree in both MCI and early Alzheimer’s disease patients compared with CN.^37^ It is worth noting that some earlier studies might not have had the sample size to detect these associations.

When comparing correlation strengths between regional SV2A DVR and cognitive measures as in our previous studies, there are a few key differences. In the 2020 study, strong correlations between hippocampal synaptic density and clinical measures (CDR-sb and episodic memory) were observed in a pooled analysis of Alzheimer’s disease and CN participants. This was also the case for a composite ROI of Alzheimer’s disease–affected regions (entorhinal, hippocampal, parahippocampal, amygdala, pre-frontal, lateral temporal, posterior cingulate cortex/precuneus, lateral parietal and lateral occipital regions). However, none of these correlations were significant within the Alzheimer’s disease or CN groups.^14^ In a follow-up study from 2022, correlations of synaptic density (as assessed by SV2A PET) and cognitive performance were evaluated within a homogeneous Alzheimer’s disease sample using a comprehensive neuropsychological testing battery.^16^ However, our current ICA approach is the first data-driven study to definitively distinguish between MCI and mild Alzheimer’s disease dementia participants with respect to synaptic density PET-derived information.

### Limitations

There are a number of limitations worth reporting. The precise role of SV2A as a pre-synaptic density marker is unconfirmed, and hence, it remains a limited indicator of synaptic density. Clinical diagnosis was made on the basis of standard clinical criteria and amyloid PET status. Biomarkers of tau pathogenesis were not included and there is no *post-mortem* validation, so this cohort could not be fully classified according to amyloid, tau, neurodegeneration criteria.^38^ This study has limited power to investigate demographic variables due to its modest sample size. It is also important to note that ICA has not been previously utilized to investigate synaptic density patterns in Alzheimer’s disease patients, and future studies with larger sample sizes may be needed to further assess and validate findings of this nature. In addition, longitudinal studies would be highly valuable to determine whether the ICA loading weights can provide information on disease progression.

## Conclusion

Multiple patterns of synaptic density relevant to underlying Alzheimer’s disease pathology were demonstrated in SV2A PET data using data-driven methodologies. Synaptic density loss in Alzheimer’s disease may occur in a systematic and network-wise manner, possibly (at least partly) according to distinct anatomical patterns. In addition, several of these patterns of synaptic density showed clear significant differences across disease stages (between MCI and mild Alzheimer’s disease dementia participants), as well as strong associations with clinical measures of cognition within the symptomatic Alzheimer’s disease group. This is a promising step forward for the application of data-driven algorithms in the assistance of clinical diagnosis and disease staging in Alzheimer’s disease.

## Supplementary Material

fcae107_Supplementary_Data

## Data Availability

In the interest of promoting transparency and reproducibility in research, we are committed to sharing the data underlying the findings presented in this manuscript. The dataset utilized for the analyses in this study will be made accessible to researchers upon formal request to the corresponding author.
