# Supplementary material for: Synaptic density patterns in early Alzheimer’s disease assessed by independent component analysis
Source: Brain Commun. 2024 Mar 26;6(2):fcae107. doi: 10.1093/braincomms/fcae107 (PMC11004947; doi:10.1093/braincomms/fcae107)
Supplement: fcae107_Supplementary_Data [file fcae107_supplementary_data.docx]

**
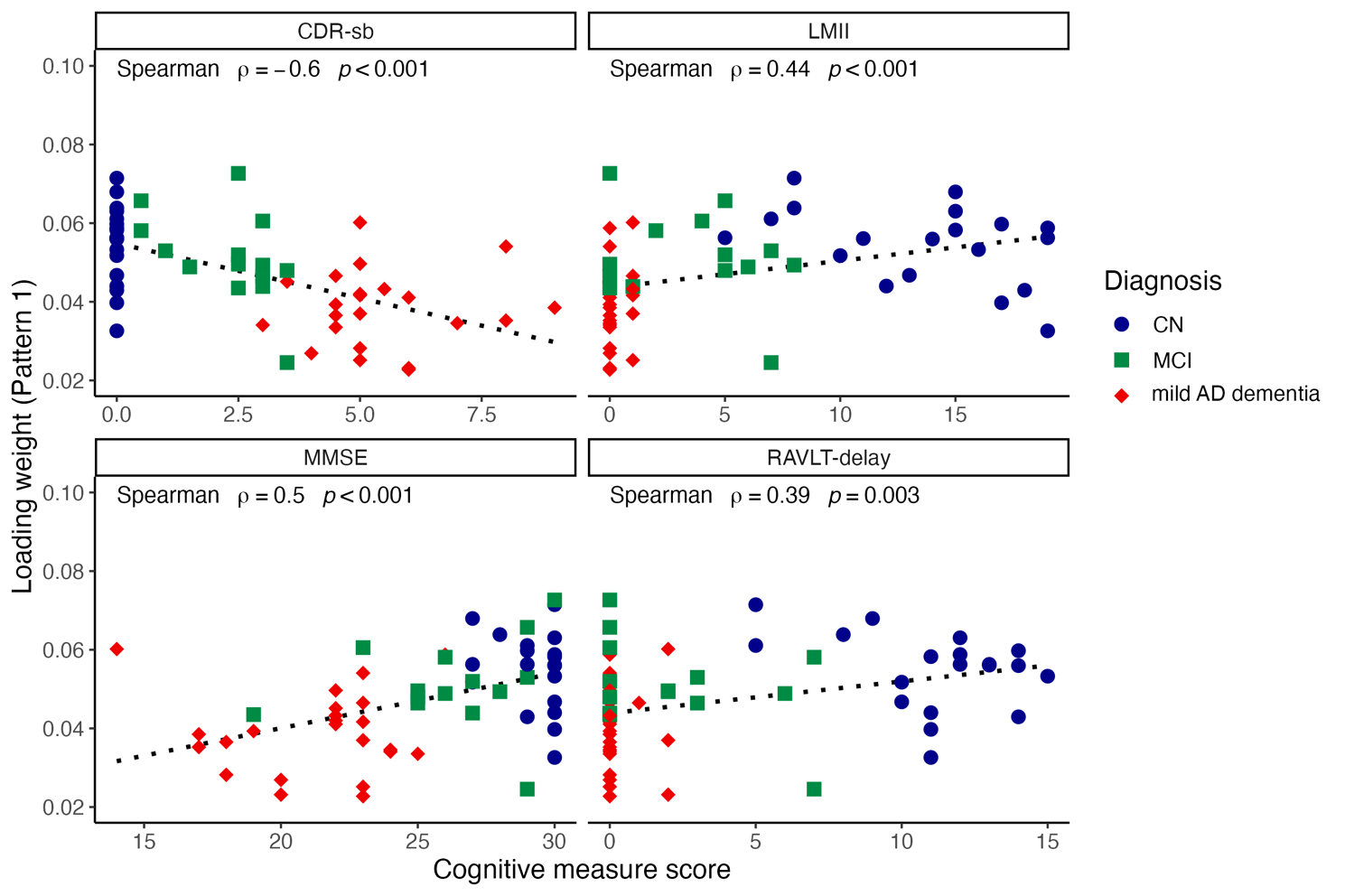
**

Supplementary Figure 1. Correlations of Pattern 1 subject loading weights and measures of cognitive and functional performance in all participants. Scatter plots depict significant relationships between loading weights and CDR-sb, LMII, MMSE, and RAVTL-delay score. Spearman’s correlations were calculated without correction for multiple comparisons, while all of these analyses survived FDR-correction for multiple comparisons. CDR-sb, clinical dementia rating sum of boxes; MMSE, Mini-Mental State Exam; LMII, logical memory II; RAVLT-delay, Rey Auditory Verbal Learning Test.

**
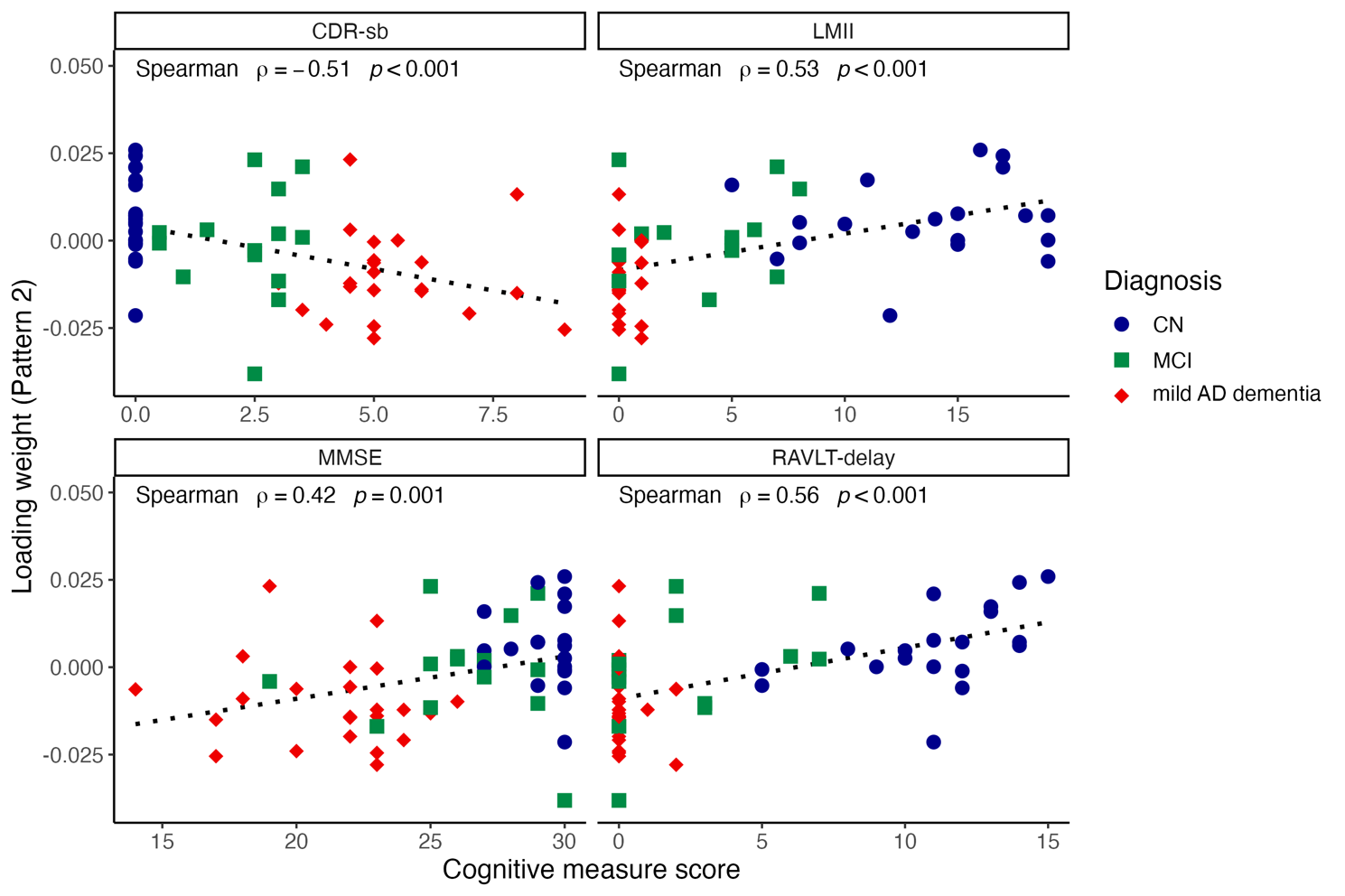
**

Supplementary Figure 2. Correlations of Pattern 2 subject loading weights and measures of cognitive and functional performance in all participants. Scatter plots depict significant relationships between loading weights and CDR-sb, LMII, MMSE, and RAVTL-delay score. Spearman’s correlations were calculated without correction for multiple comparisons, while all of these analyses survived FDR-correction for multiple comparisons. CDR-sb, clinical dementia rating sum of boxes; MMSE, Mini-Mental State Exam; LMII, logical memory II; RAVLT-delay, Rey Auditory Verbal Learning Test.

Supplementary Table 1. Participant demographics and clinical assessment outcomes.

|  | CN | MCI | Mild AD dementia | *F* / *χ*^2^ | *p* |
| --- | --- | --- | --- | --- | --- |
| Participants (n) | 19 | 14 | 24 |  |  |
| Sex (M/F) | 9/10 | 9/5 | 9/15 | 2.545 | 0.280 |
| Age (years) | 71.5 (7.6) [61-83] | 71.6 (4.2) [64-78] | 69.9 (9.2) [50-85] | 0.298 | 0.743 |
| Education (years) | 17.7 (2.1) [12-20] | 17.3 (1.9) [13-20] | 15.8 (2.4) [12-20]^**^ | 4.708 | 0.013 |
| CDR-global | 0 (0) | 0.5 (0)^****^ | 0.89 (0.21) [0.5-1]^****,††††^ | 88.29 | < 0.0001 |
| CDR-sb | 0 (0) | 2.32 (1.03) [0.5-3.5]^****^ | 5.30 (1.53) [3-9]^****,††††^ | 99.13 | < 0.0001 |
| MMSE | 29.2 (1.1) [27-30] | 26.3 (2.89) [19-30]^**^ | 21.5 (2.98) [14-30]^****, ††††^ | 52.88 | < 0.0001 |
| LMII | 13.6 (4.4) [5-19] | 3.57 (2.98)[0-8]^****^ | 0.25 (0.44) [0-1]^****,††^ | 113.50 | < 0.0001 |
| RAVLT-delay | 11.1 (2.8) [5-15] | 2.14 (2.71) [0-7]^****^ | 0.29 (0.69) [0-2]^****,††^ | 143.60 | < 0.0001 |

Data presented as mean (SD) [range]. *F* statistics and *P* values are from one-way ANOVA, *χ*^2^ and *p*-values are from *χ*^2^ significance tests. Computed *p*-values are for *χ*^2^ (categorical) or unpaired *t*-tests (continuous variables). Post hoc unpaired t-tests after one-way ANOVA (continuous variables) were Bonferroni corrected for multiple comparisons. * indicates significant group differences between either MCI and CN or mild AD dementia and CN. * p < 0.05, ** p < 0.01, *** p < 0.001, **** p < 0.0001. † denotes significant group differences between mild AD dementia and mild cognitive impairment. † p < 0.05, †† p < 0.01, ††† p < 0.001, †††† p < 0.0001. Age is at time of [^11^C]UCB-J PET scan. CDR-global, clinical dementia rating global score; CDR-sb, clinical dementia rating sum of boxes; MMSE, Mini-Mental State Exam; LMII, logical memory II; RAVLT-delay, Rey Auditory Verbal Learning Test.
